# Supplementary material for: Adaptive TreeHive: Ensemble of trees for enhancing imbalanced intrusion classification
Source: PLoS One. 2025 Sep 19;20(9):e0331307. doi: 10.1371/journal.pone.0331307 (PMC12448997; doi:10.1371/journal.pone.0331307)
Supplement: Attachment — Submitted filename: Response to Reviewers PONE-D-25-26240.pdf [file pone.0331307.s002.pdf]

Original Manuscript ID: PONE-D-25-26240

Original Article Title: **“**Adaptive TreeHive: Ensemble of Trees for Enhancing Imbalanced Intrusion Classification”

PLOS ONE

Response to Reviewers

Reviewer #1, Concern #1 (Clarify Dataset Creation and Informative Instance Selection):

Author response: Thank you for this crucial question. We define **"informative instances"** as the data points most representative of their respective classes, identified through a two-phase balancing process, updated details in the "Data Balancing" subsection of our paper. **Informative Instance Selection via Clustering:** First, for each dominant class in a dataset, we use K-Means clustering (with k=1) to find the class centroid. Instances are then ranked based on their Euclidean distance to this centroid. We select the instances closest to the centroid as the most "informative" (Ψ), as they best represent the core characteristics of that class. Scattered instances, which are farther from the centroid, are discarded to reduce noise and redundancy. This process effectively undersamples the majority of classes while preserving their essential patterns.

Balancing with SMOTE: After selecting informative instances, we address the remaining class imbalance, particularly for minority classes, by applying the Synthetic Minority Over-sampling Technique (SMOTE). SMOTE generates synthetic samples for the minority classes by interpolating between existing instances and their nearest neighbors.

This combined approach ensures our final datasets are not only balanced but are also built from high-quality, representative instances, which is vital for robust model training and reproducibility.

Reviewer #1, Concern #2 (Hyperparameter Justification.):

Author response: We appreciate the opportunity to clarify our methodology. The hyperparameters listed in

Table 2 was not chosen arbitrarily but was the result of a systematic tuning and validation process to ensure optimal performance.

Within the "Adaptive TreeHive" section, we provide justifications for these choices. For example:

- The number of feature groups
  K used in our data randomization strategy was determined to be optimal at $k=15\text{-}40$ through ablation studies, as this value offered the best trade-off between feature coverage and redundancy reduction.
- The accuracy threshold
  ϕ for selecting classifiers into our final ensemble was set to $\phi > 0.5$ based on cross-validation, as this value ensured the inclusion of reliable classifiers without sacrificing ensemble diversity.
- Tree growth parameters, such as a maximum depth ($dmax = 15$) and a minimum of 5 samples per leaf node, were set to prevent model overfitting while maintaining discriminative power.

This tuning process was integral to guiding the algorithm's optimization and achieving the reported results.

Reviewer #1, Concern #3 (Comparative Analysis with Modern Deep Learning Models.):

Author response: We thank the reviewer for this important point. We conducted a direct comparative analysis between our proposed Adaptive TreeHive and established deep learning models. This comparison is presented in our "Ablation Study" section and detailed in Table 8.

The results show that Adaptive TreeHive is highly competitive and often superior to the deep learning baselines (BiLSTM and CNN-GRU).

- Notably, our model surpassed the next-best model, CNN-GRU, by significant accuracy margins of 5.16% on UNSW-NB15, 2.61% on CIC-IDS2017, and 2.95% on CICDDoS2019.
- Beyond accuracy, we justify our focus on tree-based models by highlighting their computational efficiency. Deep learning architectures like BiLSTM and CNN-GRU are notoriously resource-intensive and require prolonged training times. In contrast, our tree-based framework is inherently more lightweight, enabling faster training and inference without specialized hardware, making it a more pragmatic and scalable solution for real-world deployment.

Reviewer #1, Concern #4 (Computational Complexity and Runtime Evaluation.):

Author response: We acknowledge the reviewer's feedback. While we did not include a specific table of execution times, our claim of reduced computational requirements is based on two core aspects of our model's design.

1. **Built-in Dimensionality Reduction:** Our methodology incorporates a unique data randomization and feature grouping strategy. As detailed in the "Adaptive TreeHive" section, each decision tree in the ensemble operates on a reduced feature subset (approximately 35% of the original features), significantly lowering the computational load for each base learner.
2. **Inherent Efficiency of Tree Ensembles:** As discussed in the "Ablation Study," our tree-based ensemble framework is inherently more lightweight and computationally efficient compared to deep learning architectures like BiLSTM and CNN-GRU, which are known to be resource-intensive and demand substantial computational overhead.

Further, the process of selecting only informative instances reduces the overall size of the training data, which directly leads to faster training times.

Reviewer #1, Concern #5 (Visualization and Result Presentation.):

Author response: We agree that strong visualizations are key to understanding model performance. In our updated manuscript, we have included detailed confusion matrices for each of the five benchmark datasets to provide these insights:

- **Figure 3:** NSL-KDD dataset
- **Figure 4:** UNSW-NB15 dataset
- **Figure 5:** CIC-IDS2017 dataset
- **Figure 6:** CSE-CIC-IDS2018 dataset
- **Figure 7:** CIC-DDoS2019 dataset

These figures are supported by an extensive, multi-paragraph analysis within the "Experimental Results" section. This analysis delves into the model's class-by-class performance, with a particular focus on its exceptional success in detecting extremely rare minority attacks, which directly highlights the model's strength and its robustness against class imbalance.

Reviewer #1, Concern #6 (Writing and Organization):

Author response: We have separated the “Limitations and future work” and “Conclusion” sections in the updated manuscript.

Reviewer #1, Concern #7 (Reproducibility):

Author response: The Experimental Setup section explains the explicit details about the hardware and software environment used in our experiments.

Reviewer #1, Concern #8 (Minor Points):

Author response: We have corrected the inconsistent usage of "base classifier" and "base classifier" and have corrected throughout the revised manuscript to ensure consistency. Furthermore, we have performed a thorough check entire paper to check all figure labels, captions, and in-text references for clarity, accuracy, and completeness.
