# Supplementary material for: Adaptive TreeHive: Ensemble of trees for enhancing imbalanced intrusion classification
Source: PLoS One. 2025 Sep 19;20(9):e0331307. doi: 10.1371/journal.pone.0331307 (PMC12448997; doi:10.1371/journal.pone.0331307)
Supplement: Attachment — Submitted filename: Comments for Authors.docx [file pone.0331307.s001.docx]

Thank you for your interesting manuscript titled “Adaptive TreeHive: Ensemble of Trees for Enhancing Imbalanced Intrusion Classification.” Your paper addresses an important topic in intrusion detection by proposing a novel ensemble approach that combines Random Forest, AdaBoost, and Bagging along with informative instance selection to mitigate data imbalance and high dimensionality issues.

To enhance the clarity, rigor, and impact of your work, please consider addressing the following comments:

1. **Clarify Dataset Creation and Informative Instance Selection**
   - You mention that large-scale balanced datasets were created using clustering and sampling methods. Please provide a more detailed description of the dataset balancing process and the criteria for informative instance selection.
   - Clearly define what you mean by “informative instances” and describe how these instances were identified in your approach. This information is critical for readers to replicate your experiments and validate your findings.
2. **Hyperparameter Justification**
   - You list the hyperparameters for your models in Table 2. Please explain how these values were chosen and whether you performed any hyperparameter tuning or cross-validation to ensure optimal performance.
3. **Comparative Analysis with Modern Deep Learning Models**
   - Although you compare Adaptive TreeHive with traditional ensemble classifiers, please discuss its performance in comparison to modern deep learning-based methods (e.g., CNN-LSTM, hybrid DL models), which are often competitive in intrusion detection tasks.
   - If direct comparisons were not conducted, please provide a brief discussion on how your method might compare to these approaches and potential reasons for focusing solely on ensemble tree-based models.
4. **Computational Complexity and Runtime Evaluation**
   - While you claim that your proposed method reduces computational power and execution time, no quantitative analysis of these claims is provided. Please include an explicit runtime or computational complexity comparison (e.g., average training/testing time) against baseline methods to substantiate this claim.
5. **Visualization and Result Presentation**
   - Consider including additional visualizations, such as ROC curves, confusion matrices, or feature importance plots, to provide deeper insights into your model’s performance and highlight its strengths or weaknesses.
6. **Writing and Organization**
   - The Introduction and Background sections are informative but could be condensed to improve clarity and flow.
   - Consider adding a dedicated section for “Limitations and Future Work” to openly discuss potential shortcomings of your method and possible avenues for future research.
7. **Reproducibility**
   - Please provide explicit details about the hardware and software environment used in your experiments (CPU, RAM, GPU, OS, etc.).
   - Consider including a link to your code or data-processing scripts (if possible), which would greatly enhance the reproducibility of your work.
8. **Minor Points**
   - Ensure consistent usage of terms like “base classifier” instead of “based classifier.”
   - Double-check figure labels, captions, and in-text references to ensure they are clear and complete.

Please address these comments in your revised manuscript to strengthen the technical rigor, reproducibility, and clarity of your work. Addressing these points will enhance the paper’s overall contribution and impact in the field of intrusion detection.
